# Subjective and objective survival prediction in mechanically ventilated critically ill patients: a prospective cohort study

**DOI:** 10.1186/s13054-023-04381-1

**Published:** 2023-04-19

**Authors:** Lucas Boeck, Hans Pargger, Peter Schellongowski, Charles-Edouard Luyt, Marco Maggiorini, Kathleen Jahn, Grégoire Muller, Rene Lötscher, Evelyne Bucher, Nadine Cueni, Thomas Staudinger, Jean Chastre, Martin Siegemund, Michael Tamm, Daiana Stolz

**Affiliations:** 1 Department of Clinical Research, University Hospital Basel, Basel, Switzerland; 2 Department of Biomedicine, University Basel, Basel, Switzerland; 3 Intensive Care Unit, Department of Acute Medicine, University Hospital Basel, Basel, Switzerland; 4 Department of Internal Medicine I, University Hospital Vienna, Vienna, Austria; 5 Médecine Intensive Réanimation, Institut de Cardiologie, Groupe Hospitalier Pitié-Salpêtrière, Assistance Publique-Hôpitaux de Paris, Sorbonne-Université, Paris, France; 6 UMRS 1166, INSERM, ICAN Institute of Cardiometabolism and Nutrition, Sorbonne Université, Paris, France; 7 Department of Internal Medicine, Intensive Care Unit, University Hospital Zürich, Zürich, Switzerland; 8 Surgical and Medical Intensive Care Medicine, Kantonsspital Baselland, Liestal, Switzerland; 9 Department of Pneumology, Medical Center, Faculty of Medicine, University of Freiburg, Freiburg, Germany

**Keywords:** Risk stratification, Personalised medicine, Critical care, Scores, Outcome, Modelling

## Abstract

**Background:**

ICU risk assessment tools, routinely used for predicting population outcomes, are not recommended for evaluating individual risk. The state of health of single patients is mostly subjectively assessed to inform relatives and presumably to decide on treatment decisions. However, little is known how subjective and objective survival estimates compare.

**Methods:**

We performed a prospective cohort study in mechanically ventilated critically ill patients across five European centres, assessed 62 objective markers and asked the clinical staff to subjectively estimate the probability of surviving 28 days.

**Results:**

Within the 961 included patients, we identified 27 single objective predictors for 28-day survival (73.8%) and pooled them into predictive groups. While patient characteristics and treatment models performed poorly, the disease and biomarker models had a moderate discriminative performance for predicting 28-day survival, which improved for predicting 1-year survival. Subjective estimates of nurses (c-statistic [95% CI] 0.74 [0.70–0.78]), junior physicians (0.78 [0.74–0.81]) and attending physicians (0.75 [0.72–0.79]) discriminated survivors from non-survivors at least as good as the combination of all objective predictors (c-statistic: 0.67–0.72). Unexpectedly, subjective estimates were insufficiently calibrated, overestimating death in high-risk patients by about 20% in absolute terms. Combining subjective and objective measures refined discrimination and reduced the overestimation of death.

**Conclusions:**

Subjective survival estimates are simple, cheap and similarly discriminative as objective models; however, they overestimate death risking that live-saving therapies are withheld. Therefore, subjective survival estimates of individual patients should be compared with objective tools and interpreted with caution if not agreeing.

*Trial registration* ISRCTN ISRCTN59376582, retrospectively registered October 31st 2013.

**Supplementary Information:**

The online version contains supplementary material available at 10.1186/s13054-023-04381-1.

## Introduction

The assessment of risk, or predictive modelling, is a fundamental strategy across nearly all medical disciplines [1–3], including respiratory conditions like community-acquired pneumonia [4], asthma [5] and COPD [6]. Similarly, risk assessment has several implications on different levels of intensive care medicine. For example, predictive modelling can evaluate population outcomes, such as intensive care unit (ICU) benchmarking, to assess whether the observed mortality matches predicted mortality. Furthermore, predictive models could be employed for personalised applications ranging from identifying patients at risk for preventive measures and clinical trials over ICU allocation strategies in resource-limited settings to severity tailored treatment regimens.

Numerous intensive care scoring systems like the Acute Physiology and Chronic Health Evaluation (APACHE) Score [7] and the Simplified Acute Physiology Score (SAPS) [8] were developed over the last decades, many of which have been validated across multiple patient populations [9]. Subsequently, several risk tools have been updated with improved algorithms and additional predictors [10, 11]. More recently, due to the surge of big data and machine learning, new well-performing algorithms have been proposed [12, 13].

Due to limited score performance and evidence supporting severity guided treatment approaches, physicians rarely use ICU scores on individual patients. Still, to inform relatives, physicians subjectively estimate patient outcomes based on clinical parameters, experience and personal factors. Whether accurate or not, these subjective estimates may affect treatment decisions, such as life support limitations [14]. While subjective clinical assessments are easy to obtain and may inform about pathophysiological features difficult to capture elsewhere, they are also prone to bias.

This prospective international study addresses the strengths and limitations of subjective and objective survival prediction markers in mechanically ventilated critically ill patients. We assess potential predictors across groups of patient characteristics, diseases and biomarkers individually, combine them in models and validate these models for predicting short- and long-term outcome. Finally, we propose how combined subjective probability estimates and objective markers synergise the strengths of different prognostic assessments.

## Methods

### Study subjects

BioVent (Biomarkers for mechanically ventilated patients) is an investigator-initiated prospective longitudinal cohort study, performed at five ICUs in Austria (Vienna General Hospital), France (Groupe Hospitalier Pitié-Salpêtrière) and Switzerland (University Hospital Basel, Kantonsspital Liestal, University Hospital Zürich). The study was registered (ISRCTN59376582), approved by the respective institutional review boards, conducted in accordance with the Declaration of Helsinki and the International Conference on Harmonisation Guidelines for Good Clinical Practice, and is reported in accordance with the STROBE statement [15, 16]. Written informed consent was obtained from the patients' legal representatives. Patients were recruited from treating physicians and study nurses. Critically ill medical and surgical patients 18 years or older at the start of mechanical ventilation (ventilated for less than 36 h) were considered for study inclusion if they were expected to be mechanically ventilated for at least 24 h or were already ventilated for 12 to maximal 36 h. Between April 2011 and February 2017, 1313 patients were screened and 961 patients were included in the study. A sample size of 450 patients in the development and validation cohort, assuming a 28-day mortality of 25%, resulted in a power of 90% to detect a minimal significant difference in c-statistic (0.6 vs. 0.5) with a two-sided alpha of 0.05. Study follow-up was finished in May 2018. The development cohort (for defining a model) and the validation cohort (for model validation) were set up as defined previously. The first 50% of patients per centre (480 patients) were included in the development cohort and the last 50% (481 patients) in the validation cohort (temporal validation). The study protocol did not provide additional information to the clinical team and did not interfere with treatment decisions.

### Predictors

At the time of study enrolment, 62 potential predictors of outcome were assessed (Additional file 1). Events or markers assessed at a later timepoint were not integrated into predictive models. Apart from the newer biomarkers pro-adrenomedullin (proADM), midregional pro-atrial natriuretic peptide (proANP) and C-terminal pro-arginine vasopressin (copeptin), for which blood was stored and analysed at study end, all measures were obtained on the same day and available to the responsible medical team. ProADM, proANP and copeptin (Brahms, Thermo Scientific Biomarkers, Hennigsdorf, Germany) were analysed as described previously [17–20]. Predictors were used to calculate the simplified acute physiologic score (SAPS) II and SOFA. Furthermore, a single nurse, junior doctor and attending physician were asked to give an estimate for 28-day survival, assigning a value on a visual analogue scale ranging from 0 (0% 28-day survival probability) to 100 (100% 28-day survival probability).

### Outcome

The outcome measures 28-day and 1-year survival, and in case of death the date of death, were obtained from the hospital, patients, family members or the patient’s general practitioner.

Details on statistical analysis are provided in the Additional file 1.

## Results

### Patients

Among 1313 critically ill mechanically ventilated patients, 961 patients were included in five European study centres (Additional file 1: Fig. S1). Patients were predominantly male (70.1%), had a mean age of 63.8 (± 15.0) years, a mean SOFA of 8.7 (± 3.4) and were admitted to medical and surgical intensive care (45.2% and 54.8%, respectively). As defined previously, the first 50% of patients per centre (*n* = 480) were included in the development cohort; the last 50% formed the validation cohort (*n* = 481). Most parameters were well balanced between the two cohorts (Additional file 1: Table S1). In total, 252 patients died within 28 days (26.2%, Additional file 1: Fig. S2).

### Prognostic markers

**Fig. 1:**
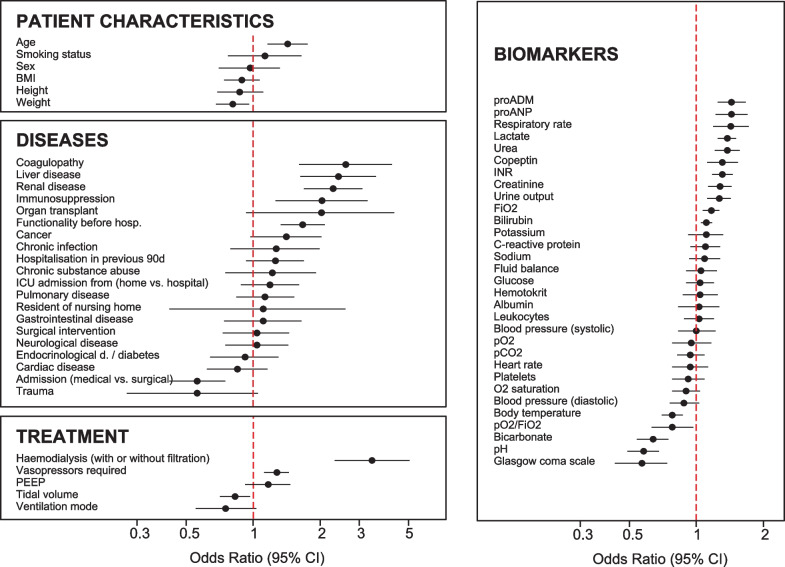
Univariate analysis of 62 markers for predicting 28-day outcome. Potential predictors were pooled into four groups: patient characteristics, diseases including acute and chronic conditions, treatment variables and biomarkers. Forest plots represent the associations of single predictors with death within 28 days (odds ratios normalised to unit and 95% confidence intervals)

To evaluate markers for survival prediction, capturing acute and chronic measures of disease and overall health, across multiple organ systems, we prospectively assessed four groups of potential outcome predictors at study inclusion; namely markers of patient characteristics (6 markers, Additional file 1: Table S2), diseases (20 markers), treatment (5 markers) and biomarkers (31 markers). Within the markers of patient characteristics, a group of disease unrelated markers, age (odds ratio [95% confidence interval]; 1.02 [1.01–1.03], *p* = 0.0008; Fig. 1) and weight (0.99 [0.98–1.00], *p* = 0.016) were predictive for 28-day survival. Stronger predictors, however, were identified in the other three groups. In the diseases group, reflecting mainly the nature of acute and chronic diseases, coagulopathy (2.61 [1.61–4.21], *p* = 0.0001) as well as renal (2.29 [1.69–3.10], *p* < 0.0001) and liver disease (2.42 [1.63–3.57], *p* < 0.0001) were the strongest predictors for outcome. Haemodialysis (with or without filtration) was the major predictor in the treatment group (3.42 [2.32–5.05], *p* < 0.0001) and the overall best single predictor for survival. The larger group of biomarkers mainly indicates the patient's acute pathophysiological condition. Next to the newer biomarkers proADM and proANP (both *p* < 0.0001), the Glasgow coma scale (0.92 [0.89–0.96], *p* < 0.0001) and especially markers of renal failure were strongly predictive for survival.

### Predictor relatedness

**Fig. 2:**
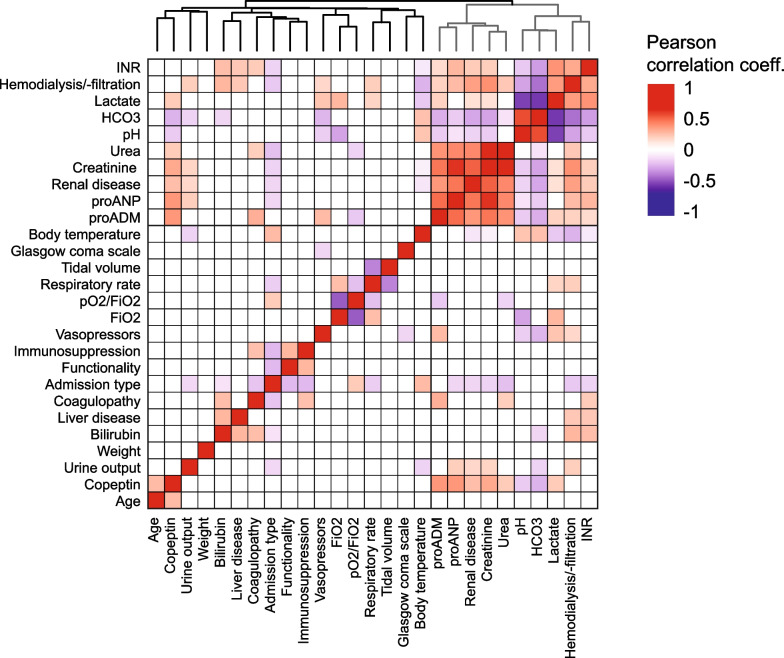
Relatedness of survival predictors. To identify predictor relatedness, a correlation matrix (Pearson correlation) of markers associated with 28-day survival was generated and ordered via hierarchical clustering (non-significant correlations are plotted in white). We observed two clusters: one cluster with highly related markers such as renal markers and newer biomarkers (generalised predictors in grey); the other cluster consisted of a diverse set of predictors with only a minor association to other markers (specialised predictors in black)

We next sought to identify the association of prognostic markers. Single markers predictive for 28-day survival were correlated and grouped with hierarchical clustering (Fig. 2). We identified two clusters of predictors. One cluster included markers poorly correlating with other predictors, presumably reflecting distinct pathophysiological features not captured by other markers (specialised predictors). Examples are markers of respiration, liver disease and the neurological state. The second cluster contained highly correlated markers of kidney function, acid–base balance and the novel biomarkers proADM and proANP, likely capturing multiple body or disease compartments with broader prognostic information (generalised predictors).

### Prognostic groups

**Fig. 3:**
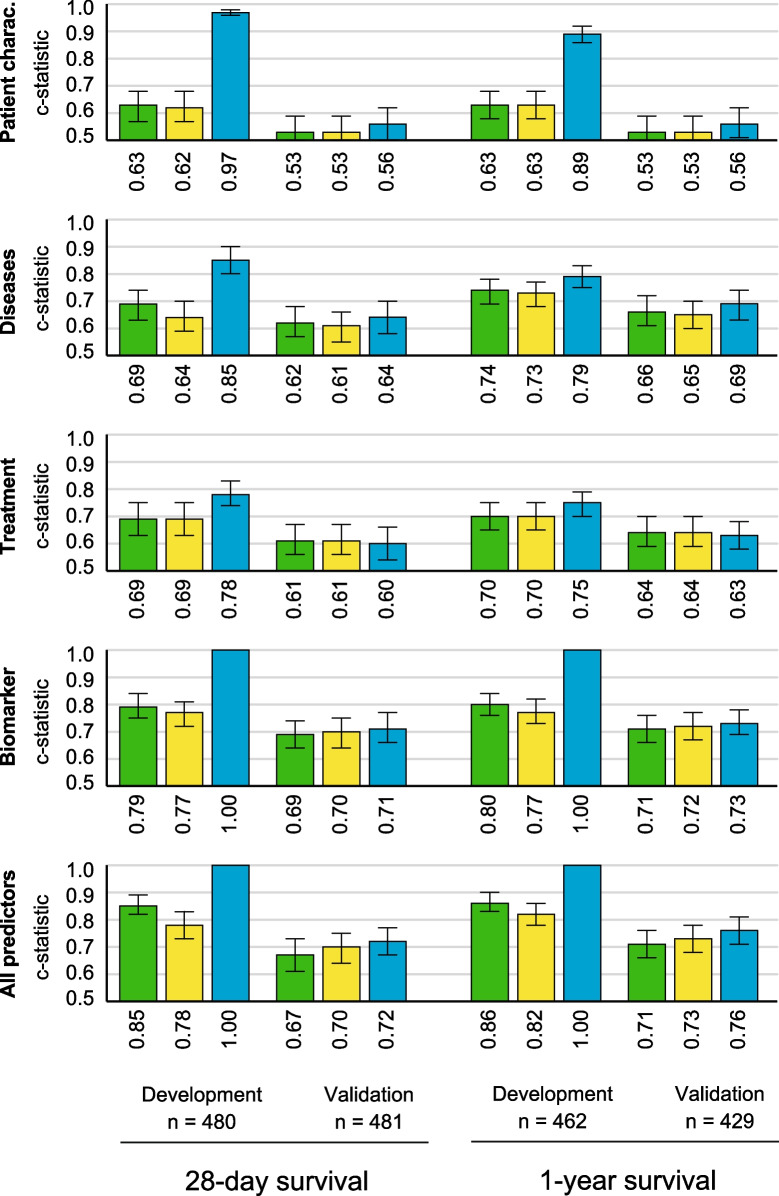
Model performance in the development and validation cohort. Different predictive models were generated using logistic regression (green), lasso regression analysis (yellow) or random forests (blue) for 28-day and 1-year survival. Models were trained in the development cohort and tested in the validation cohort. The discriminative performance was evaluated with the c-statistic (error bars reflect the 95% confidence interval). Model discrimination declined from the development to the validation cohort and improved from predicting 28-day to 1-year survival

To improve predictive performance, we combined individual objective predictors into models composed of several markers. These models were developed using three different strategies (logistic regression, lasso regression and random forest) in the development cohort. Discrimination (the ability to separate outcomes) and calibration (the agreement between observed and predicted outcomes) of the respective models were then tested in an independent prospectively assessed validation cohort (Fig. 3) [16]. In all predictive models, discriminative performance considerably declined from the development to the validation cohort. While the logistic model performed worst, overfitting was particularly evident with the random forest approach. Lasso regression provided the best calibrated models, and therefore agreement between predicted and observed probabilities (Additional file 1: Fig. S3). Models composed of markers of patient characteristics were not predictive. In other models, the discriminative performance increased from the treatment (c-statistic across different models: 0.60–0.61) and disease models (c-statistic: 0.61–0.64) to the biomarker models (c-statistic: 0.69–0.71; treatment vs. biomarker model *p* = 0.0003, disease vs. biomarker model *p* = 0.015). Combining all markers only marginally improved on top of the biomarker model (c-statistic: 0.67–0.72), which was superior to SOFA (c-statistic: 0.64 [0.60–0.69], *p* = 0.007) and similar to the performance of SAPS2 (c-statistic: 0.71 [0.67–0.75], *p* = 0.8). The performance of most models, specifically the disease model, increased for predicting 1-year survival.

### Subjective survival estimates

**Fig. 4:**
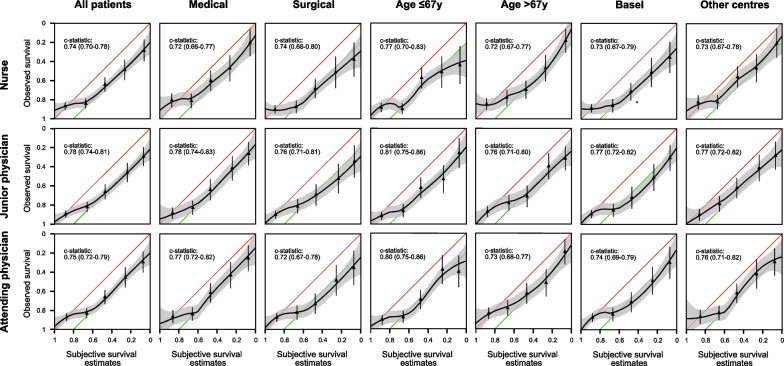
Calibration of subjective survival estimates. In order to assess calibration, subjective survival estimates were plotted against observed survival. Subjective estimates deviate from perfect calibration, especially between 0 and 60% probability of survival (red line: perfect calibration; green line: 20% absolute overestimation of death)

Considering the constraints of predictive scores, we assessed subjective survival estimates. Irrespective of training and experience, physicians discriminated considerably well between 28-day survivors and non-survivors (c-statistic junior physician: 0.78 [0.74–0.81]; c-statistic attending physician: 0.75 [0.72–0.79]). Junior physicians performed better than nurses, but we detected no difference between attending physicians and nurses (c-statistic nurse: 0.74 [0.70–0.78]; junior physician vs. nurse *p* = 0.009; attending physician vs. nurse *p* = 0.08). Regardless of the discriminative performance, we identified that the subjective survival estimates of doctors and nurses overestimated death. Specifically, between 0 and 60% probability estimates of survival, the observed survival rates were about 20% higher in absolute terms (i. e. around 20–80%, Fig. 4). This overestimation of death was similar in doctors and nurses, in medical and surgical patients and across different centres and age groups.

### Integrating subjective and objective survival assessments

**Fig. 5:**
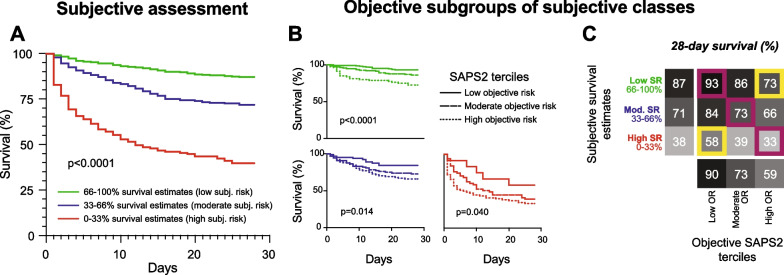
Combining subjective and objective risk assessment tools. **A** Patients were pooled into three subjective risk (SR) groups. In the moderate and high subjective risk group, survival was underestimated, i. e. death overestimated. **B** Subjective groups were further sub-grouped using SAPS2, a frequently available objective risk (OR) tool. **C** Concordant subjective and objective predictions are highlighted in purple, discordant predictions in yellow. Differences in survival were analysed with the long-rank test for trend

We then assessed if both risk assessment strategies could be combined, in particular, whether subjective estimates could be refined and recalibrated using objective assessments. We stratified patients into three subjective risk groups with 0–33%, 33–66% and 66–100% estimates of survival and further sub-grouped each group into SAPS2 risk terciles (Fig. 5). Across all three subjective risk strata, patients with a low objective risk had a significantly better outcome than patients with a high objective risk. Importantly, concordant predictions (low objective + low subjective risk or high objective + high subjective risk) supported the assessed risk, while discordant objective and subjective assessments (low objective + high subjective risk or high objective + low subjective risk) indicated prediction uncertainty.

## Discussion

This study highlights the strengths and limitations of different ICU risk assessment strategies. While objective predictive measures are generally preferable, prognostic models lack reproducibility and do not sufficiently predict outcome. Subjective estimates of the clinical staff, presumably frequently used on individual patients, perform similarly well; however, subjective assessments overestimate death, potentially affecting patient information and medical decision making. We conclude that subjective individual high-risk estimates need to be interpreted with caution and should be compared to objective risk measures.

Our first goal was to characterise different predictors individually, thereby assessing the contribution of particular pathophysiological aspects regarding outcome. We identified two groups of predictors. Predictors of the first group were poorly associated with other predictors and were considered more specific disease markers, reflecting distinct pathophysiological states. Examples are liver disorders or poor oxygenation (pO_2_/FiO_2_), which contribute to outcome if present but do not capture prognosis over a wide range of disease states. Predictors of the second group belonged to the group of highly related markers. Rather than reflecting a small disease spectrum, they address a more comprehensive range of disease entities and severity thereof. Several predictors within this group were related to kidney function, such as the requirement for dialysis, urea and urinary output. These markers were performing exceptionally well, emphasising the already well-established role of renal failure regarding outcome [21, 22]. Other predictors of this group were the newer biomarkers proADM and proANP, for which it was previously shown that they predict outcome across different acute and chronic diseases [17, 18, 20].

Single parameters do not perform sufficiently well and are commonly combined in prognostic prediction models. However, proposed models are often overly optimistic, primarily due to a small number of outcomes, a large number of predictors and feature selection approaches, also referred to as overfitting [23]. We tried to overcome these statistical limitations with several strategies. First, we used different feature selection approaches to compare their performance in order to evaluate the contribution of modelling. Furthermore, we validate all models in a predefined independent cohort using the model performance measures of discrimination and calibration. We observed that several models adequately discriminated survivors from non-survivors in the development cohort. However, despite a resampling step, the performance of all models considerably declined from the development to the validation cohort. Only the models including biomarkers or all predictors had a moderate performance in the validation cohort. In contrast, the pooled markers of patient characteristics, diseases and treatment were poorly or not predictive. This finding stresses the reliability of internal cross-validation and highlights the importance of independent validation. A particular goal of our study was to address long-term outcome. It is well known that several acute diseases contribute to mortality after the very immediate phase and hospital stay. However, new unrelated events are more likely to occur during a more extended prediction period and might restrict prediction. Surprisingly, model performance improved from 28-day to 1-year survival, indicating that the initial event, leading to mechanical ventilation, significantly contributes to mortality beyond the acute stage.

While these models provide insights into the pathophysiological compartments related to outcome, these models did not outperform SAPS2 and were insufficient to emphasise their use in individual patients. However, we argue that individual risk assessments are frequently performed to inform relatives and guide treatment decisions, such as deciding whether to initiate resuscitation procedures, life support treatments or palliative care [14]. Most commonly, ICU physicians subjectively estimate individual patient risk rather than using objective tools. We investigated whether subjective survival estimates could provide additional prognostic information. We observed that these estimates performed similarly to more complicated objective prediction models. Especially junior physicians performed well to discriminate survivors and non-survivors, whereas the performance of nurses was slightly lower. While nurses have better information on the patient's social history and life, nurses mostly do not know all details of the patient's examinations [14]. Both could negatively influence the accuracy of their estimates. Regardless of the discriminative performance, all of the clinical staff overestimated death in high-risk patients. Death was overestimated in nurses, junior doctors and attending physicians, medical and surgical patients, older and younger patients, and at different study centres. Overestimation of death is of particular concern since treatment and life support may be withheld from patients who are estimated to be at the highest risk for death [14, 24, 25].

In order to minimise misclassification and poor calibration, we combined subjective and objective prediction tools. We demonstrate that objective tools can refine subjective risk estimates. Objective measures identified patients at lower risk within different subjective groups and improved calibration of subjective assessments. While subjective low-risk estimates are relatively accurate and moderate-risk estimates presumably have a minor impact on clinical management, there should be a focus on subjective high-risk estimates. High-risk estimates need to be interpreted cautiously and if possible, compared to objective risk tools. Whereas concordant subjective and objective prediction measures may reinforce the evaluation, disagreeing results should question the assessment.

We have to report several limitations of our study. Not all eligible ICU patients have been screened throughout the study period, therefore generating the risk of selection bias. However, patient inclusion was mainly driven by available study personal and given that the study population was extremely diverse it is unlikely that a minor patient selection would have a strong effect on predictive markers. The assessed risk may have an impact on treatment and especially high-risk assessments may lead to withdrawing or withholding treatments (self-fulfilling-prophecy). Since we have no details on withholding or withdrawing treatments, we cannot exclude that risk assessments changed outcomes in single patients and may have slightly increased the performance of predictions. But importantly, if “self-fulfilling-prophecy” has occurred it would have decreased overestimation of death, and the true overestimation of death would have been even higher. There exist many risk assessment tools to benchmark ICUs. Since most scores were not routinely assessed at our study centres, comparing and assessing many predictive scores was beyond the scope and goal of this study. We focussed on mechanically ventilated patients since they are at the highest risk for death. Therefore, we do not know if our findings can be translated to non-ventilated ICU patients. However, since the study population covered many disease entities and severities, several results could also apply to the ICU population not requiring mechanical ventilation. Finally, subjective risk estimates are driven by patient and physician factors, with variable relevance. Therefore, survival perceptions probably vary across nurses and physicians and very likely across countries. We do not know if clinicians discriminate survivors from non-survivors equally well in different hospitals and if the overestimation of death occurs globally. Therefore, our findings need to be validated in larger international cohorts, including patients and clinicians of multiple backgrounds.

To summarise, we report several findings on risk assessment in mechanically ventilated ICU patients. We reveal specialised predictors and more general predictors capturing more specific or broader pathophysiological mechanisms related to outcome. We assessed different groups of objective markers for predicting short- and long-term outcomes and showed that the performance in the development cohort declines in the validation cohort, with the best combinations not outperforming SAPS2. And finally, we demonstrate that all of the clinical staff overestimates death and propose to combine subjective and objective tools to identify misclassified patients.

## Supplementary Information

**Additional file 1.** Online data supplement.

## Data Availability

Deidentified individual participant data collected during the trial will be made available upon reasonable request.
