# Supplementary material for: Subjective and objective survival prediction in mechanically ventilated critically ill patients: a prospective cohort study
Source: Crit Care. 2023 Apr 19;27:150. doi: 10.1186/s13054-023-04381-1 (PMC10114386; doi:10.1186/s13054-023-04381-1)
Supplement: Supplementary file 1 — Additional file 1. Online data supplement. [file 13054_2023_4381_MOESM1_ESM.pdf]

ONLINE DATA SUPPLEMENT

Predictors

At the time of study enrolment, potential predictors of outcome were assessed at study inclusion. Disease independent predictors included age, sex, weight, height, BMI and smoking status. Comorbidities (pulmonary, cardiac, renal, gastrointestinal, liver, cancer, neurological, endocrinological/diabetes, immunosuppression, chronic infection, chronic substance abuse, coagulopathy, trauma, organ transplant) and the fluid balance (euvolemic, hypo- or hypervolemic) were assessed by the responsible physician. It was also evaluated if the patients had a recent surgical intervention and were hospitalised within 90 days before ICU admission. The admission type (medical or surgical) and origin (home, hospital or other ICU) was documented. Several treatment parameters were assessed, namely the use of vasopressors and hemodialysis/-filtration, and mechanical ventilation settings (pressure- versus volume-controlled ventilation, tidal volume and positive end-expiratory pressure). Vital parameters (respiratory rate, O2 saturation, heart rate, blood pressure, body temperature, Glasgow Coma Scale), urine output as well as arterial blood gas parameters (pO2, pO2/FiO2, pCO2, pH, HCO3, lactate), the blood count (leukocytes, hematocrit, platelets) and other blood parameters (glucose, Na, K, albumin, creatinine, urea, bilirubin, C-reactive protein, copeptin, proANP and proADM) were recorded. The Glasgow coma scale was assessed during a sedation pause or alternatively a GCS estimate of the neurological state before sedation was used.

Statistical analysis

Descriptive statistics were expressed for continuous data as mean ±standard deviation (SD), for categorical data as numbers (%). No patient was excluded from the analysis due to missing data. Logistic regression models were used to examine the univariable association between predictors and outcome. Odds ratios (OR) and 95% confidence intervals (CI) were calculated using the Mid-P exact test. In the case of categorical predictors, a reference category was chosen. In metric or ordinal predictors, the OR represents the ratio of the odds increasing the predictor one unit. To compare different predictors, OR were IQR-normalised to units and illustrated in forest plots. The overall p-values of categorical data with two or more groups were calculated with the chi-squared test or the fisher exact test if the frequency was smaller than 5 in any group. To assess the predictor relatedness, significant predictors of 28-day survival were correlated using Pearson correlation. A correlation matrix was generated and ordered via hierarchical clustering of absolute Pearson correlation coefficients. Raw correlation coefficients were then plotted in a heatmap. To analyse groups of multiple predictors, missing values were imputed using k-nearest neighbour imputation, setting k to 5 [1]. Single objective predictors provide inadequate risk estimates. Therefore, markers are commonly combined into more complex scoring systems to improve prognostic performance [2]. However, due to optimistic model development and different patient populations, the predictive performance of these models is often not replicated. We addressed this problem using different model development approaches and independent validation thereof. Predictors were then pooled into different prediction groups. Variables with a skewed distribution (platelet number, glucose, bilirubin, urea, CRP, lactate, INR, proANP and proADM) were log-transformed. To overcome the limitations of single statistical

approaches, we applied three different prediction methods: 1) Classic logistic regression models were estimated with a generalised linear model using the logit link function. 2) Lasso models were performed as reported previously, with the tuning parameter lambda and the parameter alpha set at 0.95 [3]. 3) Random forests were applied optimising the parameter mtry [4]. In lasso and random forest, the models were trained with a 10-fold cross-validation step repeated 10 times on the development cohort and then applied on the independent validation cohort to assess its performance [5]. For these algorithms tuning parameters were chosen to optimise the AUC (c-statistic). The discrimination of each model, i. e. the power to differentiate between outcomes, was evaluated with the c-statistic ranging from 0.5 (random discrimination) to 1 (perfect discrimination). The c-statistic was estimated using DeLong's test. Comparisons between c-statistics were performed on random forest models or subjective survival estimates and compared with the DeLong's test. Calibration, the agreement of predicted vs observed outcome frequencies, was graphically assessed by plotting the predicted survival probability against the observed survival using a loess smoother. The accuracy of the calibration curve is demonstrated with five points and error bars, based on the mean of predicted probabilities, within distinct survival intervals (0-0.2, 0.2-0.4, 0.4-0.6, 0.6-0.8, 0.8-1). Survival distributions were compared with the log-rank test for trend. All statistical analyses were performed in R, version 4.0.3 (R Project for Statistical Computing). 2-sided p values less than 0.05 were considered statistically significant.

REFERENCES

1. Kowarik A, Templ M. Imputation with the R Package VIM. J Stat Softw. 2016;74.

2. Moons KGM, Altman DG, Reitsma JB, Ioannidis JPA, Macaskill P, Steyerberg EW, et al. Transparent Reporting of a multivariable prediction model for Individual Prognosis Or Diagnosis (TRIPOD): Explanation and Elaboration. Ann Intern Med. 2015;162:W1.

3. Friedman J, Hastie T, Tibshirani R. Regularization Paths for Generalized Linear Models via Coordinate Descent. J Stat Softw. 2010;33:1–22.

4. Liaw A, Wiener M. Classification and Regression by randomForest. 2002;

5. Kuhn M. Building Predictive Models in R Using the caret Package. Journal of Statistical Software. 2008;

Figure E1: Patient inclusion and follow-up.

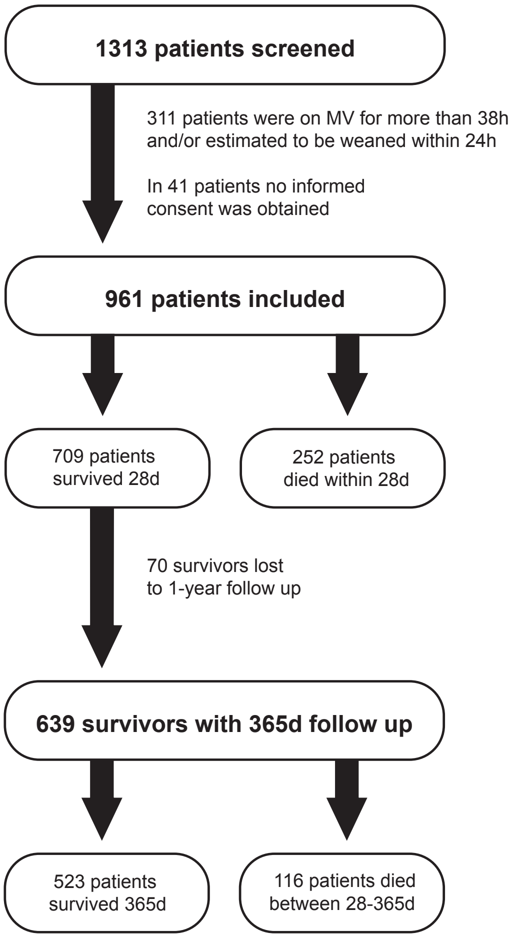

Figure E2: Mortality and treatment across study inclusion period

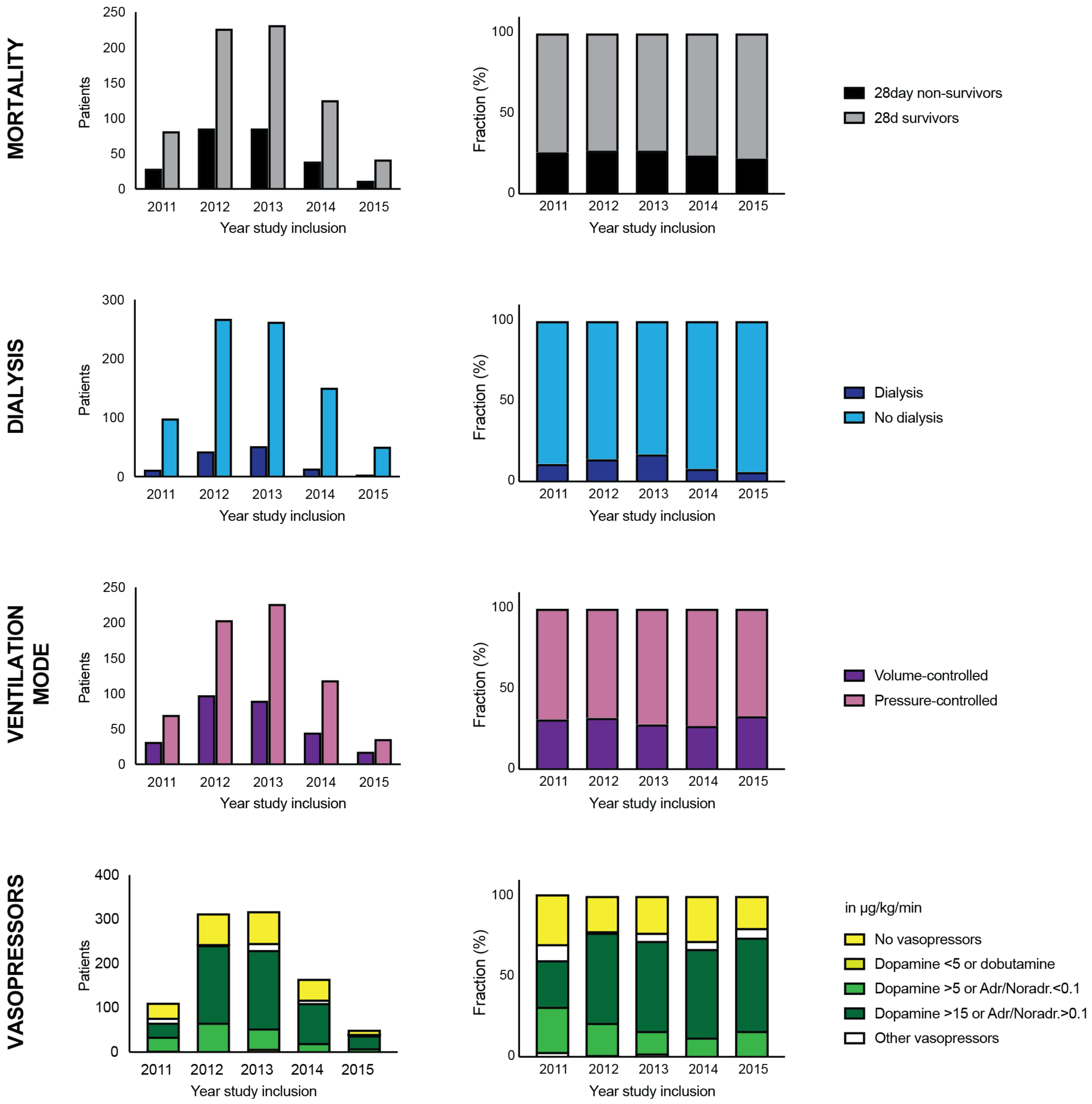

Figure E3: Calibration of objective markers. Models were trained in the development cohort and applied to the validation cohort. Calibration of different models in the validation cohort are presented. Due to poor discrimination plots of patient characteristic and treatment models are not shown.

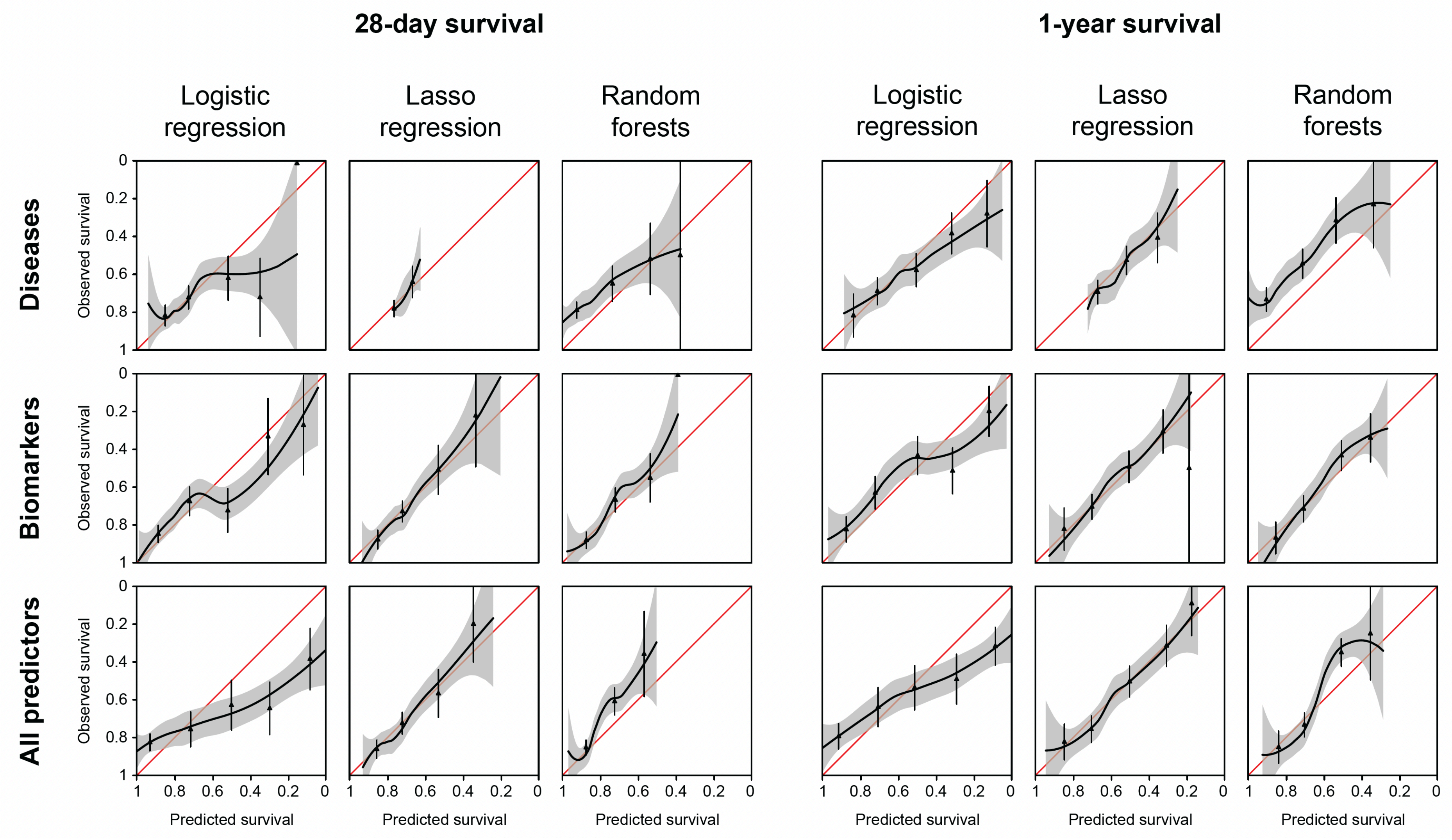

Table E1: Baseline characteristics of the total population, development and validation cohort. Categorical data are presented as numbers (%) / total numbers and continuous data as mean ±standard deviation / total numbers; * in µg/kg/min.

	All patients	Development cohort	Validation cohort
**Center**			
Basel	566 (58.9%) /961	283 (59%)	283 (58.8%)
Paris	60 (6.24%) /961	30 (6.25%)	30 (6.24%)
Liestal	19 (1.98%) /961	9 (1.88%)	10 (2.08%)
Vienna	132 (13.7%) /961	66 (13.8%)	66 (13.7%)
Zürich	184 (19.1%) /961	92 (19.2%)	92 (19.1%)
**Patient characteristics**			
Age	63.8 ±15.0 /961	63.5 ±15.2	64.1 ±14.7
Female sex	287 (29.9%) /961	147 (30.6%)	140 (29.1%)
BMI	26.9 ±5.59 /716	26.6 ±5.31	27.3 ±5.88
Weight	78.7 ±18 /940	77.4 ±16.7	79.9 ±19.1
Height	171 ±9.03 /719	171 ±8.92	171 ±9.18
Smoking status			
Never smoker	188 (31.1%) /604	82 (28.8%)	106 (33.2%)
Current smoker	227 (37.6%) /604	110 (38.6%)	117 (36.7%)
Former smoker	189 (31.3%) /604	93 (32.6%)	96 (30.1%)
**Diseases**			
Pulmonary disease	348 (36.3%) /958	184 (38.4%)	164 (34.2%)
Cardiac disease	671 (70%) /958	328 (68.5%)	343 (71.6%)
Renal disease	276 (28.8%) /958	148 (30.9%)	128 (26.7%)
Gastrointestinal disease	139 (14.5%) /958	81 (16.9%)	58 (12.1%)
Liver disease	123 (12.8%) /958	69 (14.4%)	54 (11.3%)
Cancer	159 (16.6%) /958	72 (15%)	87 (18.2%)
Neurological disease	249 (26%) /958	125 (26.1%)	124 (25.9%)
Endocrinological disease / diabetes	204 (21.3%) /957	103 (21.5%)	101 (21.1%)
Immunosuppression	79 (8.25%) /958	36 (7.52%)	43 (8.98%)
Chronic infection	98 (10.2%) /958	60 (12.5%)	38 (7.93%)
Chronic substance abuse	94 (9.81%) /958	52 (10.9%)	42 (8.77%)
Coagulopathy	76 (7.93%) /958	44 (9.19%)	32 (6.68%)
Trauma	65 (6.78%) /958	38 (7.93%)	27 (5.64%)
Organ transplant	29 (3.03%) /958	15 (3.13%)	14 (2.92%)
Surgical intervention	220 (23%) /958	102 (21.3%)	118 (24.6%)
Hospitalisation in previous 90 days	348 (36.6%) /952	163 (34.3%)	185 (38.8%)
Admission from			
Home	341 (35.5%) /960	190 (39.6%)	151 (31.5%)
Hospital	566 (59%) /960	266 (55.4%)	300 (62.5%)
Other ICU	53 (5.52%) /960	24 (5%)	29 (6.04%)
Admission type			
Medical	434 (45.2%) /960	226 (47.1%)	208 (43.3%)
Scheduled surgical	274 (28.5%) /960	114 (23.8%)	160 (33.3%)
Unscheduled surgical	252 (26.2%) /960	140 (29.2%)	112 (23.3%)
Resident of nursing home	25 (2.62%) /955	12 (2.52%)	13 (2.71%)
Functionality before hospitalisation			
Good	345 (48.3%) /715	187 (61.3%)	158 (38.5%)
Moderate	258 (36.1%) /715	87 (28.5%)	171 (41.7%)
Poor	112 (15.7%) /715	31 (10.2%)	81 (19.8%)
**Therapy**			
Haemodialysis (with or without filtration)	124 (13%) /956	57 (11.9%)	67 (14%)
Vasopressors			
No vasopressors	232 (24.1%) /961	111 (23.1%)	121 (25.2%)
Dopamine <5 or dobutamine*	12 (1.25%) /961	5 (1.04%)	7 (1.46%)
Dopamine >5 or Adr/Noradr. <0.1*	171 (17.8%) /961	102 (21.2%)	69 (14.3%)
Dopamine >15 or Adr/Noradr. >0.1*	505 (52.5%) /961	242 (50.4%)	263 (54.7%)
other vasopressors	41 (4.27%) /961	20 (4.17%)	21 (4.37%)
Pressure-controlled ventilation mode	656 (69.9%) /939	328 (71.5%)	328 (68.3%)
Tidal volume (ml)	531 ±155 /904	532 ±163	529 ±147
PEEP (cmH2O)	7.88 ±3.07 /940	7.74 ±3.2	8.02 ±2.94

	All patients	*Development cohort*	*Validation cohort*
Biomarkers			
Respiratory rate (per min)	*17.5 ±6.19 /935*	*17.2 ±6.12*	*17.8 ±6.25*
O2 saturation	*96.6 ±3.78 /942*	*96.9 ±3.48*	*96.3 ±4.03*
Heart rate (per min)	*88.4 ±18.8 /958*	*88.8 ±19.1*	*87.9 ±18.5*
Blood pressure systolic (mmHg)	*108 ±22.8 /961*	*108 ±24.7*	*109 ±20.8*
Blood pressure diastolic (mmHg)	*56.6 ±12.8 /959*	*56.7 ±14*	*56.4 ±11.4*
Body temperature (°C)	*36.7 ±1.35 /926*	*36.8 ±1.37*	*36.7 ±1.32*
Glasgow coma scale	*7.42 ±4.12 /927*	*7.72 ±4.19*	*7.13 ±4.03*
Fluid balance			
Euvolemic	*357 (37.5%) /953*	*188 (39.6%)*	*169 (35.4%)*
Hypovolemic	*173 (18.2%) /953*	*92 (19.4%)*	*81 (16.9%)*
Hypervolemic	*423 (44.4%) /953*	*195 (41.1%)*	*228 (47.7%)*
Urine output			
>1000ml	*682 (71.3%) /957*	*326 (68.1%)*	*356 (74.5%)*
500-1000ml	*155 (16.2%) /957*	*89 (18.6%)*	*66 (13.8%)*
200-500ml	*49 (5.12%) /957*	*33 (6.89%)*	*16 (3.35%)*
<200ml	*71 (7.42%) /957*	*31 (6.47%)*	*40 (8.37%)*
pO2/FiO2 (mmHg)	*267 ±113 /941*	*262 ±112*	*272 ±113*
Fraction of inspired oxygen (%)	*47.9 ±16.1 /957*	*48.1 ±16.3*	*47.8 ±15.9*
O2 (mmHg)	*115 ±39 /945*	*113 ±37*	*116 ±40*
CO2 (mmHg)	*42 ±9 /946*	*42 ±10*	*42 ±9*
pH	*7.36 ±0.08 /950*	*7.37 ±0.09*	*7.36 ±0.08*
HCO3 (mmol/L)	*23.1 ±5.09 /947*	*23.5 ±5.33*	*22.8 ±4.83*
Lactate (mmol/L)	*2.93 ±3.31 /948*	*2.92 ±3.51*	*2.94 ±3.1*
Leukocytes (G/L)	*12 ±6.18 /949*	*11.7 ±6.05*	*12.3 ±6.31*
Hematocrit (%)	*30.2 ±6.35 /953*	*30.3 ±6.44*	*30 ±6.26*
Platelets (G/L)	*178 ±123 /959*	*182 ±126*	*173 ±120*
INR	*1.31 ±0.44 /931*	*1.3 ±0.5*	*1.31 ±0.39*
Glucose (mmol/L)	*7.65 ±2.41 /948*	*7.71 ±2.71*	*7.59 ±2.07*
Na (mmol/L)	*139 ±4.61 /959*	*140 ±4.76*	*139 ±4.47*
K (mmol/L)	*4.53 ±0.64 /958*	*4.53 ±0.65*	*4.53 ±0.62*
Albumin (g/L)	*22.2 ±7.02 /922*	*21.8 ±7.15*	*22.6 ±6.87*
Creatinine (µmol/L)	*121 ±84.1 /951*	*122 ±81.1*	*120 ±87.2*
Urea (µmol/L)	*9.45 ±7.3 /956*	*9.92 ±7.71*	*8.98 ±6.83*
Bilirubin (µmol/L)	*23.5 ±43.5 /935*	*21.7 ±36.1*	*25.3 ±49.9*
CRP (mg/L)	*109 ±108 /939*	*108 ±107*	*110 ±108*
Copeptin (pmol/L)	*87.5 ±90.2 /742*	*87.6 ±92.1*	*87.4 ±88.8*
proANP (pmol/L)	*286 ±252 /739*	*311 ±296*	*267 ±211*
proADM (nmol/L)	*3.55 ±3.41 /731*	*3.92 ±4.19*	*3.27 ±2.64*
Scores			
SOFA	*8.7 ±3.43 /877*	*8.65 ±3.33*	*8.75 ±3.53*
SAPS2	*46.4 ±14.9 /845*	*46.2 ±15.3*	*46.6 ±14.5*

Table E2: Single markers for predicting 28-day and 1-year survival. Categorical data are presented as numbers (%) / total numbers and continuous data as mean ±standard deviation / total numbers; * in µg/kg/min. P-values and odds ratios [95% confidence intervals] of metric data and single categorical predictors were calculated using the Mid-P exact test. Overall p-values (ov.) of categorical data were calculated with the chi-squared or fisher exact test, as appropriate.

	All patients	28-day survivors	28-day non-survivors	Odds ratio	P	All patients with 1-year outcome	1-year survivors	1-year non-survivors	Odds ratio	P
**Patient characteristics**										
Age	63.8 ±15.0 /961	62.8 ±14.9	66.5 ±14.9	1.02 [1.01-1.03]	0.0008	64.0 ±15.0 /891	62.2 ±15.3	66.5 ±14.1	1.02 [1.01-1.03]	<0.0001
Female sex	287 (29.9%) /961	213 (30%)	74 (29.4%)	0.97 [0.70-1.32]	0.8445	263 (29.5%) /891	154 (29.4%)	109 (29.6%)	1.01 [0.75-1.35]	0.9540
BMI	26.9 ±5.59 /716	27.1 ±5.52	26.5 ±5.8	0.98 [0.95-1.01]	0.2219	26.9 ±5.62 /651	27.1 ±5.34	26.6 ±5.98	0.98 [0.96-1.01]	0.2680
Weight	78.7 ±18 /940	79.5 ±18.1	76.3 ±17.6	0.99 [0.98-1.00]	0.0158	78.4 ±17.6 /873	79.3 ±16.8	77.2 ±18.6	0.99 [0.99-1.00]	0.0800
Height	171 ±9.03 /719	171 ±9.21	170 ±8.48	0.99 [0.97-1.01]	0.2671	171 ±9 /654	171 ±9.5	170 ±8.28	0.99 [0.98-1.01]	0.4131
Smoking status					0.7474 (ov.)					0.2567 (ov.)
Never smoker	188 (31.1%) /604	146 (31.9%)	42 (28.8%)	Ref.	Ref.	171 (30.7%) /557	106 (32.9%)	65 (27.7%)	Ref.	Ref.
Current smoker	227 (37.6%) /604	169 (36.9%)	58 (39.7%)	1.19 [0.76-1.89]	0.4501	206 (37%) /557	120 (37.3%)	86 (36.6%)	1.17 [0.77-1.77]	0.4639
Former smoker	189 (31.3%) /604	143 (31.2%)	46 (31.5%)	1.12 [0.69-1.81]	0.6491	180 (32.3%) /557	96 (29.8%)	84 (35.7%)	1.42 [0.93-2.19]	0.1028
**Diseases**										
Pulmonary disease	348 (36.3%) /958	251 (35.6%)	97 (38.5%)	1.13 [0.84-1.53]	0.4057	325 (36.5%) /890	169 (32.3%)	156 (42.5%)	1.55 [1.17-2.04]	0.0020
Cardiac disease	671 (70%) /958	501 (71%)	170 (67.5%)	0.85 [0.62-1.16]	0.2991	625 (70.2%) /890	372 (71.1%)	253 (68.9%)	0.90 [0.67-1.21]	0.4824
Renal disease	276 (28.8%) /958	170 (24.1%)	106 (42.1%)	2.29 [1.69-3.10]	<0.0001	255 (28.7%) /890	103 (19.7%)	152 (41.4%)	2.88 [2.14-3.89]	<0.0001
Gastrointestinal disease	139 (14.5%) /958	100 (14.2%)	39 (15.5%)	1.11 [0.74-1.65]	0.6078	126 (14.2%) /890	65 (12.4%)	61 (16.6%)	1.40 [0.96-2.05]	0.0799
Liver disease	123 (12.8%) /958	70 (9.92%)	53 (21%)	2.42 [1.63-3.57]	<0.0001	115 (12.9%) /890	45 (8.6%)	70 (19.1%)	2.50 [1.68-3.76]	<0.0001
Cancer	159 (16.6%) /958	108 (15.3%)	51 (20.2%)	1.41 [0.97-2.03]	0.0749	146 (16.4%) /890	68 (13%)	78 (21.3%)	1.80 [1.26-2.58]	0.0012
Neurological disease	249 (26%) /958	182 (25.8%)	67 (26.6%)	1.04 [0.75-1.44]	0.7979	232 (26.1%) /890	143 (27.3%)	89 (24.3%)	0.85 [0.62-1.16]	0.3024
Endocrinological disease / diabetes	204 (21.3%) /957	153 (21.7%)	51 (20.2%)	0.92 [0.64-1.30]	0.6321	186 (20.9%) /889	101 (19.3%)	85 (23.2%)	1.26 [0.91-1.74]	0.1710
Immunosuppression	79 (8.25%) /958	47 (6.66%)	32 (12.7%)	2.04 [1.26-3.27]	0.0042	72 (8.09%) /890	25 (4.78%)	47 (12.8%)	2.91 [1.77-4.90]	<0.0001
Chronic infection	98 (10.2%) /958	68 (9.63%)	30 (11.9%)	1.27 [0.79-1.99]	0.3102	90 (10.1%) /890	38 (7.27%)	52 (14.2%)	2.10 [1.35-3.29]	0.0009
Chronic substance abuse	94 (9.81%) /958	66 (9.35%)	28 (11.1%)	1.22 [0.75-1.92]	0.4198	85 (9.55%) /890	46 (8.8%)	39 (10.6%)	1.23 [0.78-1.93]	0.3629
Coagulopathy	76 (7.93%) /958	41 (5.81%)	35 (13.9%)	2.61 [1.61-4.21]	0.0001	67 (7.53%) /890	20 (3.82%)	47 (12.8%)	3.67 [2.16-6.46]	<0.0001
Trauma	65 (6.78%) /958	54 (7.65%)	11 (4.37%)	0.56 [0.27-1.05]	0.0700	64 (7.19%) /890	52 (9.94%)	12 (3.27%)	0.31 [0.15-0.57]	0.0001
Organ transplant	29 (3.03%) /958	17 (2.41%)	12 (4.76%)	2.03 [0.93-4.31]	0.0751	28 (3.15%) /890	10 (1.91%)	18 (4.9%)	2.62 [1.21-6.02]	0.0141
Surgical intervention	220 (23%) /958	161 (22.8%)	59 (23.4%)	1.04 [0.73-1.45]	0.8389	207 (23.3%) /890	120 (22.9%)	87 (23.7%)	1.04 [0.76-1.43]	0.7902
Hospitalisation in previous 90 days	348 (36.6%) /952	247 (35.1%)	101 (40.6%)	1.26 [0.93-1.69]	0.1285	326 (37%) /882	164 (31.7%)	162 (44.4%)	1.72 [1.30-2.27]	0.0001
Admission from					0.3886 (ov.)					0.0509 (ov.)
Home	341 (35.5%) /960	259 (36.5%)	82 (32.7%)	Ref.	Ref.	308 (34.6%) /890	189 (36.1%)	119 (32.4%)	Ref.	Ref.
Hospital	566 (59%) /960	414 (58.4%)	152 (60.6%)	1.16 [0.85-1.59]	0.3508	535 (60.1%) /890	314 (60%)	221 (60.2%)	1.12 [0.84-1.49]	0.4479
Other ICU	53 (5.52%) /960	36 (5.08%)	17 (6.77%)	1.50 [0.78-2.78]	0.2196	47 (5.28%) /890	20 (3.82%)	27 (7.36%)	2.14 [1.15-4.04]	0.0166
Admission type					0.0001 (ov.)					<0.0001 (ov.)
Medical	434 (45.2%) /960	294 (41.5%)	140 (55.8%)	Ref.	Ref.	380 (42.7%) /890	179 (34.2%)	201 (54.8%)	Ref.	Ref.
Scheduled surgical	274 (28.5%) /960	224 (31.6%)	50 (19.9%)	0.47 [0.32-0.67]	<0.0001	268 (30.1%) /890	186 (35.6%)	82 (22.3%)	0.39 [0.28-0.55]	<0.0001
Unscheduled surgical	252 (26.2%) /960	191 (26.9%)	61 (24.3%)	0.67 [0.47-0.95]	0.0251	242 (27.2%) /890	158 (30.2%)	84 (22.9%)	0.47 [0.34-0.66]	<0.0001
Resident of nursing home	25 (2.62%) /955	18 (2.56%)	7 (2.79%)	1.11 [0.42-2.60]	0.8233	24 (2.71%) /885	13 (2.5%)	11 (3.01%)	1.21 [0.52-2.77]	0.6449
Functionality before hospitalisation					<0.0001 (ov.)					<0.0001 (ov.)
Good	345 (48.3%) /715	279 (52.2%)	66 (36.5%)	Ref.	Ref.	317 (47.7%) /664	227 (56.6%)	90 (34.2%)	Ref.	Ref.
Moderate	258 (36.1%) /715	188 (35.2%)	70 (38.7%)	1.57 [1.07-2.31]	0.0210	241 (36.3%) /664	137 (34.2%)	104 (39.5%)	1.91 [1.34-2.73]	0.0003
Poor	112 (15.7%) /715	67 (12.5%)	45 (24.9%)	2.83 [1.78-4.51]	<0.0001	106 (16%) /664	37 (9.23%)	69 (26.2%)	4.68 [2.94-7.54]	<0.0001

	All patients	28-day survivors	28-day non-survivors	Odds ratio	P	All patients with 1-year outcome	1-year survivors	1y non-survivors	Odds ratio	P
Therapy										
Haemodialysis (with or without filtration)	*124 (13%) /956*	*62 (8.78%)*	*62 (24.8%)*	*3.42 [2.32-5.05]*	*<0.0001*	*116 (13.1%) /886*	*37 (7.1%)*	*79 (21.6%)*	*3.60 [2.39-5.52]*	*<0.0001*
Vasopressors					*0.0002 (ov.)*					*<0.0001 (ov.)*
No vasopressors	*232 (24.1%) /961*	*196 (27.6%)*	*36 (14.3%)*	*Ref.*	*Ref.*	*223 (25%) /891*	*169 (32.3%)*	*54 (14.7%)*	*Ref.*	*Ref.*
*Dopamine <5 or dobutamine**	*12 (1.25%) /961*	*7 (0.99%)*	*5 (1.98%)*	*3.89 [1.07-13.2]*	*0.0404*	*11 (1.23%) /891*	*3 (0.57%)*	*8 (2.17%)*	*8.01 [2.18-39.6]*	*0.0015*
*Dopamine >5 or Adr/Noradr. <0.1**	*171 (17.8%) /961*	*121 (17.1%)*	*50 (19.8%)*	*2.24 [1.38-3.67]*	*0.0010*	*150 (16.8%) /891*	*81 (15.5%)*	*69 (18.8%)*	*2.66 [1.71-4.16]*	*<0.0001*
*Dopamine >15 or Adr/Noradr. >0.1**	*505 (52.5%) /961*	*358 (50.5%)*	*147 (58.3%)*	*2.23 [1.50-3.38]*	*<0.0001*	*468 (52.5%) /891*	*248 (47.4%)*	*220 (59.8%)*	*2.77 [1.95-3.98]*	*<0.0001*
other vasopressors	*41 (4.27%) /961*	*27 (3.81%)*	*14 (5.56%)*	*2.82 [1.32-5.86]*	*0.0084*	*39 (4.38%) /891*	*22 (4.21%)*	*17 (4.62%)*	*2.41 [1.18-4.89]*	*0.0167*
Pressure-controlled ventilation mode	*656 (69.9%) /939*	*498 (71.4%)*	*158 (65.3%)*	*0.75 [0.55-1.03]*	*0.0746*	*595 (68.5%) /869*	*354 (69.3%)*	*241 (67.3%)*	*0.91 [0.68-1.22]*	*0.5415*
Tidal volume (ml)	*531 ±155 /904*	*538 ±157*	*510 ±147*	*1.00 [1.00-1.00]*	*0.0174*	*534 ±158 /835*	*555 ±164*	*507 ±146*	*1.00 [1.00-1.00]*	*<0.0001*
PEEP (cmH2O)	*7.88 ±3.07 /940*	*7.8 ±3.03*	*8.1 ±3.18*	*1.03 [0.98-1.08]*	*0.1965*	*7.87 ±3.08 /870*	*7.84 ±3.05*	*7.92 ±3.12*	*1.01 [0.96-1.05]*	*0.7327*
Biomarkers										
Respiratory rate (per min)	*17.5 ±6.19 /935*	*17.1 ±6.22*	*18.8 ±5.93*	*1.05 [1.02-1.07]*	*0.0002*	*17.6 ±6.21 /866*	*16.8 ±6.19*	*18.6 ±6.1*	*1.05 [1.02-1.07]*	*0.0001*
O2 saturation	*96.6 ±3.78 /942*	*96.7 ±3.77*	*96.3 ±3.81*	*0.97 [0.94-1.01]*	*0.1499*	*96.7 ±3.85 /873*	*96.9 ±3.85*	*96.2 ±3.82*	*0.95 [0.92-0.99]*	*0.0080*
Heart rate (per min)	*88.4 ±18.8 /958*	*88.6 ±18.4*	*87.7 ±20*	*1.00 [0.99-1.01]*	*0.4975*	*88.4 ±18.7 /889*	*88 ±17.8*	*88.9 ±20*	*1.00 [1.00-1.01]*	*0.4736*
Blood pressure systolic (mmHg)	*108 ±22.8 /961*	*108 ±22.9*	*109 ±22.6*	*1.00 [0.99-1.01]*	*0.9642*	*108 ±22.9 /891*	*107 ±22.9*	*109 ±23*	*1.00 [1.00-1.01]*	*0.2613*
Blood pressure diastolic (mmHg)	*56.6 ±12.8 /959*	*57 ±13.1*	*55.4 ±11.8*	*0.99 [0.98-1.00]*	*0.1042*	*56.5 ±13 /889*	*57 ±13.4*	*55.7 ±12.3*	*0.99 [0.98-1.00]*	*0.1577*
Body temperature (°C)	*36.7 ±1.35 /926*	*36.8 ±1.24*	*36.4 ±1.55*	*0.80 [0.72-0.88]*	*<0.0001*	*36.8 ±1.33 /857*	*36.9 ±1.2*	*36.5 ±1.48*	*0.82 [0.74-0.91]*	*0.0001*
Glasgow coma scale	*7.42 ±4.12 /927*	*7.76 ±4.03*	*6.45 ±4.24*	*0.92 [0.89-0.96]*	*<0.0001*	*7.63 ±4.09 /861*	*8.2 ±3.81*	*6.81 ±4.34*	*0.92 [0.89-0.95]*	*<0.0001*
Fluid balance					*0.0663 (ov.)*					*0.047 (ov.)*
Euvolemic	*357 (37.5%) /953*	*273 (38.8%)*	*84 (33.6%)*	*Ref.*	*Ref.*	*325 (36.8%) /883*	*192 (36.9%)*	*133 (36.6%)*	*Ref.*	*Ref.*
Hypovolemic	*173 (18.2%) /953*	*116 (16.5%)*	*57 (22.8%)*	*1.60 [1.07-2.38]*	*0.0233*	*151 (17.1%) /883*	*76 (14.6%)*	*75 (20.7%)*	*1.42 [0.96-2.10]*	*0.0752*
Hypervolemic	*423 (44.4%) /953*	*314 (44.7%)*	*109 (43.6%)*	*1.13 [0.81-1.57]*	*0.4723*	*407 (46.1%) /883*	*252 (48.5%)*	*155 (42.7%)*	*0.89 [0.66-1.20]*	*0.4358*
Urine output					*<0.0001 (ov.)*					*<0.0001 (ov.)*
>1000ml	*682 (71.3%) /957*	*538 (76.1%)*	*144 (57.6%)*	*Ref.*	*Ref.*	*639 (72%) /887*	*421 (80.8%)*	*218 (59.6%)*	*Ref.*	*Ref.*
500-1000ml	*155 (16.2%) /957*	*106 (15%)*	*49 (19.6%)*	*1.73 [1.17-2.53]*	*0.0065*	*139 (15.7%) /887*	*64 (12.3%)*	*75 (20.5%)*	*2.26 [1.56-3.29]*	*<0.0001*
200-500ml	*49 (5.12%) /957*	*27 (3.82%)*	*22 (8.8%)*	*3.04 [1.66-5.51]*	*0.0004*	*43 (4.85%) /887*	*12 (2.3%)*	*31 (8.47%)*	*4.94 [2.54-10.2]*	*<0.0001*
<200ml	*71 (7.42%) /957*	*36 (5.09%)*	*35 (14%)*	*3.62 [2.19-6.00]*	*<0.0001*	*66 (7.44%) /887*	*24 (4.61%)*	*42 (11.5%)*	*3.36 [2.00-5.79]*	*<0.0001*
pO2/FiO2 (mmHg)	*267 ±113 /941*	*272 ±109*	*253 ±120*	*1.00 [1.00-1.00]*	*0.0232*	*269 ±114 /872*	*281 ±112*	*252 ±115*	*1.00 [1.00-1.00]*	*0.0002*
Fraction of inspired oxygen (%)	*47.9 ±16.1 /957*	*46.8 ±14.8*	*51.1 ±18.8*	*1.02 [1.01-1.02]*	*0.0004*	*48.2 ±15.9 /887*	*46.8 ±13.8*	*50.4 ±18.3*	*1.01 [1.01-1.02]*	*0.0010*
O2 (mmHg)	*115 ±39 /945*	*115 ±38*	*113 ±41*	*1.00 [1.00-1.00]*	*0.6289*	*116 ±39 /876*	*119 ±39*	*112 ±39*	*0.99 [0.99-1.00]*	*0.0048*
CO2 (mmHg)	*42 ±9 /946*	*42 ±9*	*41 ±10*	*0.99 [0.98-1.01]*	*0.4219*	*42 ±9 /878*	*42 ±9*	*42 ±10*	*1.00 [0.98-1.01]*	*0.6970*
pH	*7.36 ±0.08 /950*	*7.37 ±0.07*	*7.33 ±0.1*	*0.00 [0.00-0.02]*	*<0.0001*	*7.36 ±0.08 /881*	*7.37 ±0.07*	*7.34 ±0.1*	*0.02 [0.00-0.09]*	*<0.0001*
HCO3 (mmol/L)	*23.1 ±5.09 /947*	*23.7 ±4.67*	*21.6 ±5.83*	*0.92 [0.89-0.94]*	*<0.0001*	*23.1 ±5.14 /878*	*23.7 ±4.48*	*22.2 ±5.85*	*0.94 [0.92-0.97]*	*<0.0001*
Lactate (mmol/L)	*2.93 ±3.31 /948*	*2.43 ±2.42*	*4.35 ±4.74*	*1.18 [1.13-1.24]*	*<0.0001*	*3 ±3.41 /879*	*2.42 ±2.24*	*3.82 ±4.44*	*1.15 [1.09-1.21]*	*<0.0001*
Leukocytes (G/L)	*12 ±6.18 /949*	*12 ±5.84*	*12.1 ±7.11*	*1.00 [0.98-1.03]*	*0.7232*	*12 ±6.08 /879*	*11.9 ±5.57*	*12.2 ±6.75*	*1.01 [0.99-1.03]*	*0.4846*
Hematocrit (%)	*30.2 ±6.35 /953*	*30.1 ±6.12*	*30.3 ±6.96*	*1.01 [0.98-1.03]*	*0.6573*	*30 ±6.19 /883*	*30.1 ±5.96*	*29.8 ±6.52*	*0.99 [0.97-1.02]*	*0.5381*
Platelets (G/L)	*178 ±123 /959*	*180 ±123*	*171 ±124*	*1.00 [1.00-1.00]*	*0.3314*	*178 ±124 /889*	*178 ±111*	*177 ±141*	*1.00 [1.00-1.00]*	*0.9183*
INR	*1.31 ±0.44 /931*	*1.26 ±0.37*	*1.45 ±0.58*	*2.48 [1.74-3.53]*	*<0.0001*	*1.31 ±0.45 /862*	*1.25 ±0.38*	*1.4 ±0.52*	*2.27 [1.56-3.31]*	*<0.0001*
Glucose (mmol/L)	*7.65 ±2.41 /948*	*7.63 ±2.23*	*7.73 ±2.87*	*1.02 [0.96-1.08]*	*0.5677*	*7.63 ±2.41 /880*	*7.58 ±2.29*	*7.7 ±2.58*	*1.02 [0.97-1.08]*	*0.4821*
Na (mmol/L)	*139 ±4.61 /959*	*139 ±4.29*	*140 ±5.41*	*1.02 [0.99-1.05]*	*0.2641*	*139 ±4.68 /889*	*139 ±4.24*	*140 ±5.24*	*1.01 [0.99-1.04]*	*0.3245*
K (mmol/L)	*4.53 ±0.64 /958*	*4.52 ±0.6*	*4.57 ±0.73*	*1.13 [0.91-1.42]*	*0.2731*	*4.54 ±0.64 /889*	*4.5 ±0.61*	*4.59 ±0.69*	*1.25 [1.01-1.54]*	*0.0372*
Albumin (g/L)	*22.2 ±7.02 /922*	*22.2 ±6.9*	*22.3 ±7.36*	*1.00 [0.98-1.02]*	*0.7814*	*21.9 ±6.87 /857*	*21.8 ±6.63*	*22 ±7.21*	*1.01 [0.99-1.03]*	*0.5588*
Creatinine (µmol/L)	*121 ±84.1 /951*	*114 ±81.2*	*140 ±89.2*	*1.00 [1.00-1.01]*	*0.0001*	*121 ±83.6 /882*	*106 ±67.9*	*142 ±98.4*	*1.01 [1.00-1.01]*	*<0.0001*
Urea (µmol/L)	*9.45 ±7.3 /956*	*8.69 ±7.37*	*11.6 ±6.65*	*1.05 [1.03-1.07]*	*<0.0001*	*9.41 ±7.02 /886*	*7.82 ±6.06*	*11.7 ±7.64*	*1.10 [1.07-1.13]*	*<0.0001*
Bilirubin (µmol/L)	*23.5 ±43.5 /935*	*19.9 ±32.8*	*33.7 ±64.2*	*1.01 [1.00-1.01]*	*0.0004*	*23.1 ±41.8 /873*	*18.9 ±29.5*	*29.2 ±54.3*	*1.01 [1.00-1.01]*	*0.0017*
CRP (mg/L)	*109 ±108 /939*	*106 ±104*	*116 ±118*	*1.00 [1.00-1.00]*	*0.2224*	*108 ±107 /870*	*96.9 ±94.4*	*123 ±121*	*1.00 [1.00-1.00]*	*0.0004*
Copeptin (pmol/L)	*87.5 ±90.2 /742*	*80.8 ±84.5*	*107 ±103*	*1.00 [1.00-1.00]*	*0.0008*	*88.9 ±90.9 /694*	*79.7 ±84.6*	*103 ±98.1*	*1.00 [1.00-1.00]*	*0.0014*
proANP (pmol/L)	*286 ±252 /739*	*260 ±232*	*362 ±291*	*1.00 [1.00-1.00]*	*<0.0001*	*286 ±254 /691*	*227 ±161*	*376 ±331*	*1.00 [1.00-1.00]*	*<0.0001*
proADM (nmol/L)	*3.55 ±3.41 /731*	*3.16 ±2.88*	*4.72 ±4.45*	*1.13 [1.08-1.18]*	*<0.0001*	*3.49 ±3.3 /684*	*2.73 ±2.3*	*4.64 ±4.13*	*1.24 [1.17-1.32]*	*<0.0001*
Scores										
SOFA	*8.7 ±3.43 /877*	*8.25 ±3.28*	*10 ±3.51*	*1.17 [1.11-1.22]*	*<0.0001*	*8.62 ±3.43 /821*	*7.79 ±3.16*	*9.83 ±3.47*	*1.20 [1.15-1.26]*	*<0.0001*
SAPS2	*46.4 ±14.9 /845*	*43.5 ±13.9*	*54.8 ±14.5*	*1.06 [1.04-1.07]*	*<0.0001*	*45.9 ±15.1 /789*	*40.9 ±13.3*	*53.2 ±14.6*	*1.06 [1.05-1.08]*	*<0.0001*
